# Radiomic Gradient in Peritumoural Tissue of Liver Metastases: A Biomarker for Clinical Practice? Analysing Density, Entropy, and Uniformity Variations with Distance from the Tumour

**DOI:** 10.3390/diagnostics14141552

**Published:** 2024-07-18

**Authors:** Francesco Fiz, Elisa Maria Ragaini, Sara Sirchia, Chiara Masala, Samuele Viganò, Marco Francone, Lara Cavinato, Ettore Lanzarone, Angela Ammirabile, Luca Viganò

**Affiliations:** 1 Nuclear Medicine Unit, Department of Diagnostic Imaging, Ente Ospedaliero “Ospedali Galliera”, 16128 Genoa, Italy; 2 Department of Nuclear Medicine and Clinical Molecular Imaging, University Hospital, 72076 Tübingen, Germany; 3 Department of Biomedical Sciences, Humanitas University, Pieve Emanuele, 20072 Milan, Italy; elisamaria.ragaini@st.hunimed.eu (E.M.R.); marco.francone@hunimed.eu (M.F.); angela.ammirabile@gmail.com (A.A.); 4 Department of Management, Information and Production Engineering, University of Bergamo, 24129 Bergamo, Italy; s.sirchia@studenti.unibg.it (S.S.); c.masala@studenti.unibg.it (C.M.); ettore.lanzarone@unibg.it (E.L.); 5 MOX Laboratory, Department of Mathematics, Politecnico di Milano, 20133 Milan, Italy; viganosamuele2005@gmail.com (S.V.); lara.cavinato@polimi.it (L.C.); 6 Department of Diagnostic and Interventional Radiology, IRCCS Humanitas Research Hospital, 20089 Milan, Italy; 7 Hepatobiliary Unit, Department of Minimally Invasive General & Oncologic Surgery, Humanitas Gavazzeni University Hospital, 24125 Bergamo, Italy

**Keywords:** radiomics, liver neoplasms, tomography, X-ray computed, surgical oncology, margins of excision

## Abstract

The radiomic analysis of the tissue surrounding colorectal liver metastases (CRLM) enhances the prediction accuracy of pathology data and survival. We explored the variation of the textural features in the peritumoural tissue as the distance from CRLM increases. We considered patients with hypodense CRLMs >10 mm and high-quality computed tomography (CT). In the portal phase, we segmented (1) the tumour, (2) a series of concentric rims at a progressively increasing distance from CRLM (from one to ten millimetres), and (3) a cylinder of normal parenchyma (Liver-VOI). Sixty-three CRLMs in 51 patients were analysed. Median peritumoural HU values were similar to Liver-VOI, except for the first millimetre around the CRLM. Entropy progressively decreased (from 3.11 of CRLM to 2.54 of Liver-VOI), while uniformity increased (from 0.135 to 0.199, *p* < 0.001). At 10 mm from CRLM, entropy was similar to the Liver-VOI in 62% of cases and uniformity in 46%. In small CRLMs (≤30 mm) and responders to chemotherapy, normalisation of entropy and uniformity values occurred in a higher proportion of cases and at a shorter distance. The radiomic analysis of the parenchyma surrounding CRLMs unveiled a wide halo of progressively decreasing entropy and increasing uniformity despite a normal radiological aspect. Underlying pathology data should be investigated.

## 1. Introduction

Radiomics is a mathematical technique that unveils invisible-to-eye patterns within a targeted area or volume of medical images [1,2]. This information is expected to mirror the biological characteristics of the tissue, as confirmed by the increasing number of papers demonstrating the association between textural features and tumour pathological data and prognosis [3,4,5,6]. One of the most fascinating applications of radiomics is the capability to detect microscopic alterations reflecting the earliest phases of pathological processes within a macroscopically normal area. Considering the liver, some preliminary reports demonstrated that the radiomic analysis of the healthy parenchyma may predict the occurrence of liver metastases before their radiological appearance [7]. Similarly, the textural features of the seemingly normal hepatic peritumoural tissue have been associated with tumour aggressiveness and patients’ prognosis [8,9,10]. 

In recent years, the study of the liver–tumour interface has gained momentum, especially for colorectal liver metastases (CRLM). The pathological features of the peritumoural tissue (e.g., neoplastic growth pattern, micrometastases, immune infiltrate, etc.) are not visible in standard imaging but dictate prognosis [11,12] and justify the need for a surgical margin—a layer of healthy parenchyma around the tumour—to prevent local recurrence risk [13,14,15]. A non-invasive assessment of the liver–tumour interface could drive clinical and surgical strategies. 

Fiz et al. demonstrated that in the portal phase of contrast-enhanced computed tomography (CT), the peritumoural tissue of CRLMs (a 5 mm thick rim) appears radiologically identical to the normal liver (with similar density) but exhibits a higher entropy and a lower homogeneity, with values similar to the tumour [16]. The present analysis aimed to elucidate the variation of the textural feature as the distance from the tumour increases and to identify the clinical parameters associated with those modifications. Results could address future research on radiomics and their correlation with pathology data.

## 2. Materials and Methods

### 2.1. Study Design

All consecutive patients with histologically proven CRLM undergoing a liver resection at the Humanitas Research Hospital in Rozzano between 1 January 2010 and 30 April 2022, and those undergoing resection or ablation at the Humanitas Gavazzeni University Hospital in Bergamo between 1 May 2022 and 1 December 2022 were considered for the study. 

The primary end goal of the study was to analyse the variation of three radiomic indices (mean Hounsfield Units (HU), entropy log2, and uniformity) in the peritumoural tissue as the distance from the tumour increases. The standard reference was the value of the radiomic indices extracted from the normal liver parenchyma, distant from the tumour. The secondary end goal was to analyse the impact of the tumour size and the response to preoperative chemotherapy on the indices’ variation. 

The study is retrospective and based on a per-lesion analysis. Up to three CRLMs per patient were considered.

According to the study endpoints, we used the following inclusion criteria: (1) age ≥ 18 years; (2) CT scan performed at the authors’ centre and available for radiomic analysis; (3) high-quality CT, i.e., absence of artefacts, complete scan of the whole liver, slice thickness ≤ 5 mm, and patient positioning with raised arms; (4) adequate portal phase of the CT; (5) CRLM ≥ 10 mm; (6) hypodense tumour clearly identifiable in the CT and with clear margins; and (7) CRLM with a 10 mm thick rim of radiologically normal peritumoural liver parenchyma present for at least 50% of the tumour circumference in each slide without major intrahepatic vessels in that area. The CRLMs having a diameter <10 mm were excluded from the study because the analysis of a 10 mm thick rim surrounding the metastasis was considered too large for millimetric lesions. We used the following exclusion criteria: (1) patients with local recurrence after a locoregional treatment (ablation or chemo-embolisation); (2) patients undergoing repeated surgery for recurrent disease (both local and non-local recurrence); (3) patients with preoperative portal vein embolisation; (4) patients with complete radiological response at the end of systemic therapy. In patients with multiple CRLMs, a distance > 30 mm between the analysed CRLM and the other nodules was mandatory.

The study was performed according to the declaration of Helsinki and its subsequent amendments. The protocol was reviewed and approved by the local ethics committee (Humanitas Research Hospital Ethics Committee, protocol 988/22, date of approval 30 December 2022). Because of the retrospective nature of the investigation, the need to obtain specific consent was waived.

### 2.2. Image Acquisition

The two participating centres had a standardised protocol for CT scan acquisition across the study period (tube voltage 120 KvP, tube current 100–434 mA, helical acquisition mode, slice thickness 2.5–5 mm). The protocol included a pre-contrast phase, an automatically bolus-triggered arterial phase, a portal phase (60–80″ contrast administration delay), and an equilibrium (late) phase (3 min). The dose of contrast agent (Iomeron 300 mg/mL; Bracco Diagnostics, Milan, Italy) was administered according to patients’ body weight (range 90–150 mL). Bolus tracking over the abdominal aorta near the celiac axis (threshold at 120 Hounsfield units) was used to time the arterial acquisition.

### 2.3. Image Segmentation

**Figure 1:**
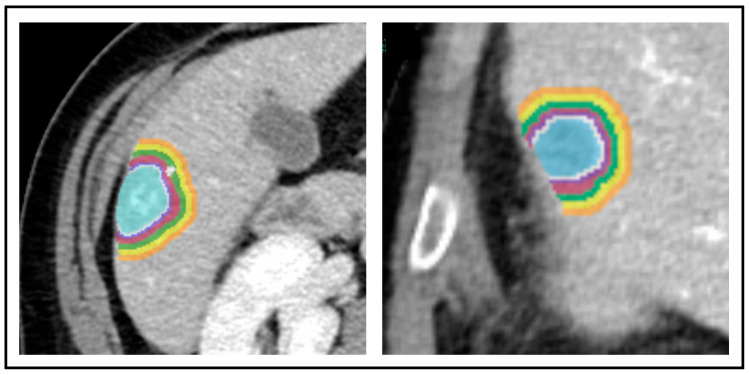
Two patients with colorectal liver metastases. The segmentation of the tumour and peritumoural rims was performed. The colours represent the different layers.

The study relies on a per-lesion analysis, and in patients with multiple CRLMs, up to three tumours were considered. All included metastases had to respect the inclusion criteria, i.e., size > 10 mm, adequate peritumoural tissue, and distance from other CRLMs ≥30 mm. The segmentation was performed using the LifeX software V. 7.4 (www.lifexsoft.org, accessed on 7 July 2023) [17]. Firstly, the lesion was identified in the portal phase and manually segmented (Tumour-VOI). Secondly, multiple concentric volumes were automatically created around the tumour to identify the tissue at 1, 2, 3–4, 5–6, 7–8, and 9–10 mm from the CRLM. The first volume (1 mm peritumoural rim) was created by an automatic expansion of the original Tumour-VOI, i.e., generating a 1 mm thick rim around the segmented tumour. The following volume was generated by applying an automatic expansion to the last-generated volume (i.e., to the 1 mm VOI). The other volumes were automatically generated by repeating the same procedure as the last-generated VOI. The first two rims were 1 mm thick (1 and 2 mm), while the subsequent ones were 2 mm thick (3–4, 5–6, 7–8, and 9–10 mm). After generating all the rims, a manual correction of the VOIs was performed to remove any included extrahepatic structures or intrahepatic vessels. Figure 1 shows two examples of segmentation. The normal liver parenchyma was analysed by drawing a virtual biopsy of the non-tumoural parenchyma (a cylinder with a basis of 10 mm diameter and a height of 25 mm), as previously reported [18]. The virtual biopsy was a distance of at least 30 mm from the tumour and was considered the standard reference. 

In the authors’ centre, the segmentation technique has been standardised since the inception of radiomic studies on liver tumours, ensuring high precision and consistency across studies and operators. Two expert medical imaging specialists with significant expertise in hepatobiliary imaging and artificial intelligence protocols (AA and FF) performed the segmentations, as follows: one performed the segmentation and the other checked for adequacy. A third expert radiologist (MF) was involved in the case of discordance.

### 2.4. Imaging Pre-Processing and Radiomic Analysis

Before processing, all images were normalised to ensure consistent parameters across images acquired on different devices, as follows: (1) spatial resampling with voxel size output (x, y, and z): 0.46 × 0.46 × 3 mm; (2) intensity discretisation: 400 grey levels, size of bin: 10; (3) intensity rescaling (absolute bounds): −1000 to 3000. Textural processing was set in 3D mode.

The radiomic features were extracted by the LifeX software, version 6.3. A separate extraction was performed for every single VOI. According to the previous study by Fiz et al. [16], we focused the present analysis on three radiomic indices: mean Hounsfield Units (HU), entropy log2, and uniformity. These three parameters represent the average density of the tissue, the inherent uncertainty/randomness in the grey-level intensities of an image, and the similarity of the voxel values within the studied area, respectively. The indices were computed according to the IBSI standard [19]. 

### 2.5. Clinical Parameters

For the present analysis, the following clinical data were collected: age and sex of the included patients, number and size of CRLMs, and preoperative systemic therapy details. If preoperative chemotherapy was administered, we registered the number of lines, the last regimen, the association with targeted therapies, the number of cycles, and the radiological response at the last restaging. The radiological response of CRLM to the preoperative systemic therapy was classified according to the RECIST v1.1 criteria [20] and morphological criteria in patients receiving anti-VEGF treatment [21]. The radiological response was systematically reviewed by two expert medical imaging specialists (AA and FF).

### 2.6. Statistical Analyses

The present study is a retrospective analysis. Categorical variables are expressed in terms of occurrences and percentages, while continuous variables are expressed in terms of mean value and range/standard deviation or median value and interquartile range (IQR). Categorical variables were compared using the chi-square test or Fisher’s exact test as appropriate. The Shapiro–Wilk test was used to measure continuous variables and determine distribution normality. Continuous variables were then compared with the parametric (unpaired T-test) test if normally distributed or the non-parametric (Mann–Whitney U test) test otherwise. The absolute values of the three radiomic indices were compared among different VOIs (Tumour-VOI, 6-Rim-VOIs, and the virtual biopsy). Using the radiomic indices extracted from the virtual biopsy as the standard reference, we analysed the percentage variation of index values across different VOIs. Given that variability in the values of radiomic indices may occur within the same tissue across different segmented areas, we have defined any value of the analysed radiomic index falling within a range of ±10% of the value observed in the virtual biopsy as equivalent to the virtual biopsy. Subgroup analyses were performed according to the tumour size (10–30 mm vs. >30 mm) and the administration of chemotherapy (no chemotherapy vs. responders to chemotherapy). A *p*-value < 0.05 was considered significant for all tests. In cases of multiple comparisons, the Bonferroni correction was applied.

## 3. Results

### 3.1. Characteristics of the Population

During the study period, 816 patients with CRLMs were considered. Among them, 448 had a CT scan available for radiomic analysis, but only 213 had a high-quality CT performed at the authors’ centre. Of those 213 patients, 162 were excluded because they did not fulfil the study inclusion criteria (CRLM size, radiological appearance, adequacy of peritumoural tissue, and previous loco-regional treatment). Overall, 63 CRLMs in 51 patients were analysed.

Table 1 summarises the patients’ characteristics. The cohort had a median age of 67 years (range, 40–76) and included 14 (27%) females. The mean tumour size was 30 mm (range 10–110), and six CRLMs (10%) were larger than 50 mm. Twenty-nine patients out of 51 (57%) had chemotherapy, 22 with a partial response and seven with a stable disease.

### 3.2. Density (HU_mean)

**Figure 2:**
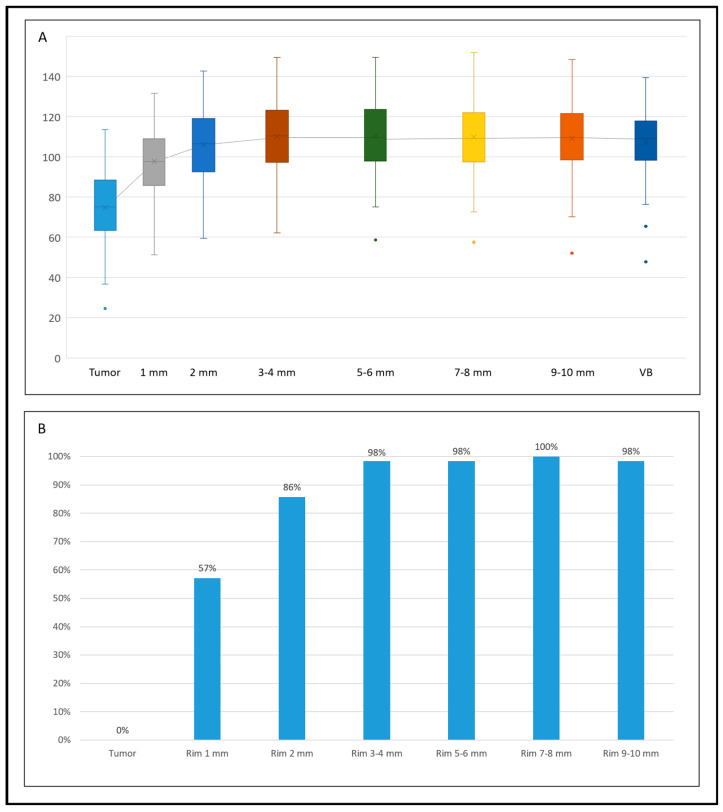
The trend of HU_mean values within different VOIs, from the tumour to the virtual biopsy (VB). (**A**) Box plots of the absolute values in the different VOIs (top); (**B**) percentage of tumours with a HU_mean value equal (±10%) to the non-tumoural parenchyma (bottom).

According to the inclusion criteria, all tumours were hypodense in comparison with the normal liver parenchyma, as confirmed by the HU_mean value of the tumour (median 75.2, IQR 63.5–88.4) in comparison with the HU_mean value of the virtual biopsy (109.2, IQR 98.3–117.8, *p* < 0.001, Table 2 and Table 3). The tissue of the first rim around the tumour (1 mm) had a HU_mean value higher than the tumour (97.7, IQR 85.7–109.0, *p* < 0.001) and lower than the non-tumoural parenchyma (*p* = 0.002 vs. virtual biopsy, Table 2 and Table 3). The rim at 2 mm from the tumour had a density higher than the 1 mm rim (106.5, IQR 92.5–119.1, *p* = 0.006), but not significantly different from the virtual biopsy. In all further rims, density values were similar to the normal parenchyma and higher than the tumour. Overall, a HU_mean value equal to the normal liver parenchyma (i.e., ±10% of the value of virtual liver biopsy) was reached in 57% of cases at 1 mm from the tumour, 86% at 2 mm, and 98% at 3 mm (Figure 2 and Supplementary Figure S1).

### 3.3. Entropy

**Figure 3:**
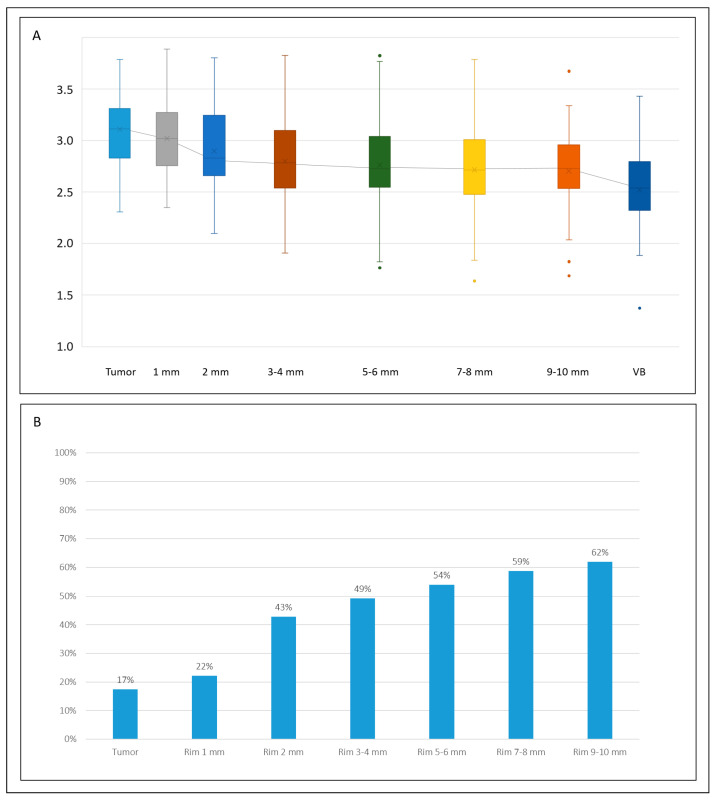
The trend of entropy values within different VOIs, from the tumour to the virtual biopsy (VB). (**A**) Box plots of the absolute values in the different VOIs (top); (**B**) percentage of tumours with an entropy value equal (±10%) to the non-tumoural parenchyma (bottom).

The median entropy value ranged from 3.11 (IQR 2.83–3.31) of the tumour to 2.54 (IQR 2.32–2.80) of the virtual biopsy (*p* < 0.001), with a median difference of 0.50 units (IQR 0.30–0.85) and 19.1% (IQR 12.1–36.5%, Table 2 and Table 3). The 1 mm rim had an entropy value similar to the tumour (median 3.02, IQR 2.76–3.27, *p* = 0.153) and higher than the normal liver parenchyma (*p* < 0.001, Table 2 and Table 3). The remaining rims had progressively decreasing entropy values, all lower than the tumour and higher than the virtual biopsy (*p* ≤ 0.007 for all—significance threshold according to the Bonferroni correction). This was evident also for the most external VOI: at 9–10 mm, the median entropy value was 2.73 (IQR 2.54–2.96), still higher than the normal parenchyma (*p* = 0.003, median difference +7.8%). As shown in Figure 3 and Supplementary Figure S2, the proportion of cases with an entropy value equal to the normal liver parenchyma (i.e., ±10% of the value of virtual biopsy) progressively increased. It passed from 22% at 1 mm to 43% at 2 mm and to 49% at 3–4 mm, but reached only 62% at 9–10 mm.

### 3.4. Uniformity

**Figure 4:**
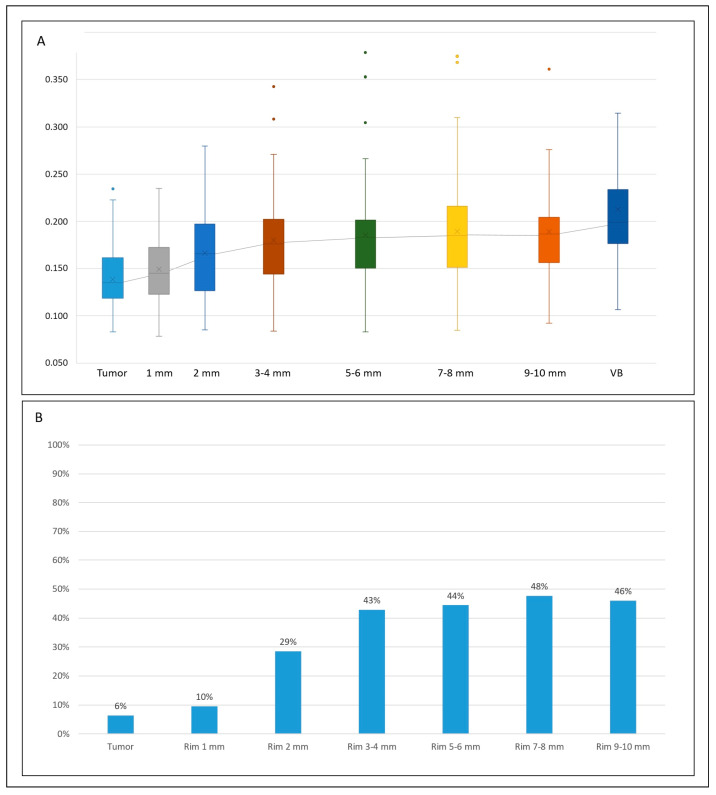
The trend of uniformity values within different VOIs, from the tumour to the virtual biopsy (VB). (**A**) Box plots of the absolute values in the different VOIs (top); (**B**) percentage of tumours with a uniformity value equal (±10%) to the non-tumoural parenchyma (bottom).

The median uniformity value increased from 0.135 (IQR 0.119–0.161) in the tumour to 0.199 (IQR 0.176–0.234) in the normal parenchyma (*p* < 0.001), with a median difference of 0.059 units (IQR 0.098–0.037) and 33.1% (IQR 19.4–46.9%, Table 2 and Table 3). The 1 mm rim had a uniformity value similar to the tumour (*p* = 0.091) and lower than the normal liver parenchyma (*p* < 0.001, Table 2 and Table 3). The remaining rims had progressively increasing uniformity values, all higher than the tumour and lower than the virtual biopsy (*p* ≤ 0.007 for all—significance threshold according to the Bonferroni correction). This was evident also for the outermost VOI: at 9–10 mm, the median uniformity value was 0.187 (IQR 0.157–0.204), still lower than the normal parenchyma (*p* = 0.007, median difference −10.4%). As shown in Figure 4 and Supplementary Figure S3, the proportion of patients with a uniformity value equal to the normal liver parenchyma (i.e., ±10%) rapidly increased in the first rims (10% at 1 mm, 29% at 2 mm, and 43% at 3–4 mm), but then reached a plateau (46% at 9–10 mm).

### 3.5. Impact of Tumour Size

**Figure 5:**
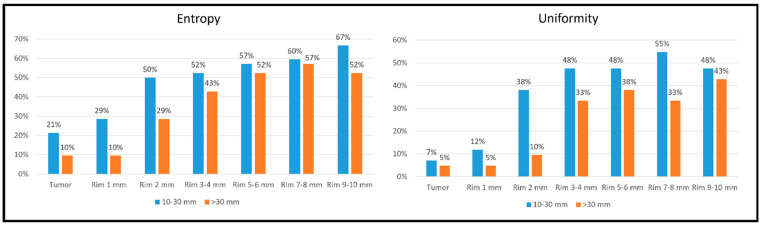
Percentage of tumours with entropy and uniformity values equal (±10%) to the non-tumoural parenchyma (virtual biopsy) within different VOIs (tumour and rims) according to the tumour size (10–30 mm vs. >30 mm).

We split the tumours according to their size: 42 CRLMs with a 10–30 mm diameter vs. 21 with a diameter larger than 30 mm. In both groups, the entropy and uniformity values had the same trend: entropy diminished as the distance from the tumour increased, and uniformity increased up to 3–4 mm and then reached a plateau (Supplementary Figure S4). However, small tumours (10–30 mm) had an earlier normalisation of the indices than the large ones (>30 mm): at 2 mm, entropy and uniformity values were equal to those of the virtual biopsy (±10%) in 50 vs. 29% of cases (*p* = 0.105) and 38 vs. 10% (*p* = 0.020), respectively (Figure 5).

### 3.6. Impact of Chemotherapy

**Figure 6:**
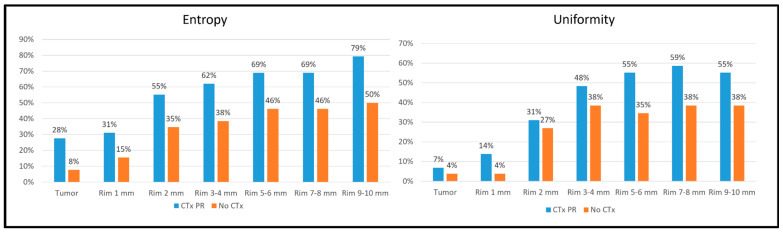
Percentage of tumours with entropy and uniformity values equal (±10%) to the non-tumoural parenchyma (virtual biopsy) within different VOIs (tumour and rims) according to the administration of preoperative chemotherapy (no chemotherapy vs. chemotherapy with partial response). CTx: chemotherapy; PR: partial response.

We split the tumours according to the pre-treatment chemotherapy administration: 26 CRLMs without chemotherapy vs. 29 with a partial response to chemotherapy (eight tumours with stable disease were excluded). In both groups, the entropy and uniformity values had the same trend: entropy diminished as the distance from the tumour increased; uniformity increased up to 3–4 mm and then reached a plateau (Supplementary Figure S5). The tumours with a partial response during chemotherapy had an earlier normalisation of the entropy and uniformity values than those without chemotherapy (Figure 6). At 1 mm, the entropy and uniformity values were equal to those of the virtual biopsy (±10%) in 31 vs. 15% of cases and 14 vs. 4%, respectively. This difference persisted even in the furthest rim (at 9–10 mm, 79 vs. 50% of cases for entropy, *p* = 0.023, and 55 vs. 38% for uniformity, *p* = 0.215).

## 4. Discussion

The present data demonstrate that the tissue surrounding CRLMs has modifications invisible to the naked eye that extend up to one centimetre around the visible lesion. While tissue density returns to normal values within the first two peritumoural millimetres, entropy and homogeneity remain altered over a wider distance, gradually normalising as the distance from the tumour increases. Small tumours and those having received effective therapy have a normalisation of the radiomic indices in a shorter distance.

Radiomic analysis has proven to be highly capable of predicting tumour pathology data and patients’ prognosis for primary and metastatic liver tumours [4,5,6]. The textural analysis of the peritumoural tissue has further improved the performance of the predictive models [8,9,10]. Such evidence sounds logical: the parenchyma surrounding the neoplasm is the niche of relevant biomarkers (micrometastases and immune infiltrate) and the battlefield for tumour progression (neoangiogenesis, tumour regrowth after chemotherapy, and replacement growth pattern) [11,12,13,15,23,24]. In addition, liver tumours should always be removed with an adequate surgical margin—a layer of peritumoural healthy parenchyma—to prevent the local recurrence risk [25,26,27]. In this scenario, we explored the variation of textural features in the peritumoural liver parenchyma as the distance from the tumour increased. Present results may address future research on radiomics and their correlation with pathology data.

Before discussing the results, two methodological aspects should be considered. Firstly, even if standard textural analyses provide several features, we focused on three radiomic indices, i.e., density, entropy, and uniformity. The first one corresponds to the radiological appearance of the parenchyma, while the other two have a strong association with pathology details and prognosis of liver metastases [28,29,30]. Fiz et al. considered the same indices when analysing the radiomic variation of the peritumoural tissue after intravenous contrast administration [16]. Secondly, we not only considered the absolute values of the features but also their relative values in comparison with the non-tumoural parenchyma remote from the neoplasm. This approach helped overcome any variability among tumours that could have biased the interpretation of the data.

Tissue density rapidly normalised within the first two millimetres. This confirms the normal appearance of the peritumoural tissue at standard radiological evaluation. Conversely, entropy and homogeneity had a progressive normalisation across a wider distance, with uniformity reaching a plateau after 3–4 mm. The observed variation of the radiomic indices implies that the textural pattern of the tumour extends beyond its visible borders. Biologically, malignant neoplasms are characterised by uncontrolled, unordered proliferation, as opposed to normal tissue, where proliferative processes are tightly regulated. We focused on two radiomics indices representing order (homogeneity) and complexity (entropy). Homogeneity (also called uniformity or energy) is the sum of the squares of all intensity values within a VOI; the larger the number of the single intensity values, the less the homogeneity. Entropy reflects the information necessary to encode the voxel distribution in the target VOI: more convoluted distribution patterns will yield higher entropy scores. In our previous publication, we found that tumour lesions had lower homogeneity and higher entropy than healthy tissue [16]. In the present analysis, we found that these two indices show a definite gradient along the peritumoural layers. The alteration of entropy and uniformity could be related to different pathology features, such as peritumoural tumour regrowth after chemotherapy, tumour budding in CRLM with a replacement pattern, and lymphatic, vascular, or perineural infiltration; the presence of immune-related inflammation is also possible. Among other hypotheses, alternating areas of tumoural neoangiogenesis and hypoxic tissues could explain the observed pattern.

At one centimetre from the tumour, entropy and homogeneity still had values that differed from those of normal liver tissue in about 40% and 50% of patients, respectively. To date, no study recommends a surgical margin >10 mm because a 10 mm margin is enough to guarantee a very low local recurrence risk (<5%), and a wider one is difficult to obtain [25,27]. It is unclear whether the present results might justify the residual local recurrence risk even in patients who underwent tumour excision with a wide margin (10 mm). Further analyses are needed. 

The subgroup analyses provided additional interesting insights. The small metastases (≤30 mm) had a normalisation of the indices within a shorter distance than the large ones (>30 mm). Of note, to date, no study has demonstrated a clear association between the tumour size and adequate width of the surgical margin. Even more relevantly, the patients showing response to chemotherapy had less radiomic alterations in the peritumoural tissue than untreated patients: their entropy value became equal to that of the normal parenchyma within 1 mm in one-third of cases and within 10 mm in more than 80% (vs. 15 and 50% of untreated patients, respectively). The latter result is in line with the clinical evidence that responders may require a smaller surgical margin than non-responders or untreated patients, possibly due to the chemo-induced reduction of micro-metastases or increase in desmoplastic pattern [31,32,33,34,35].

The present results might help bridge the gap between radiomics and its clinical applicability. The progressive modification of the textural features in the peritumoural tissue strongly confirms their effectiveness as relevant biomarkers. Some preliminary evidence demonstrated the capability of radiomics to predict the tumour growth pattern [36,37]. If textural analysis correlates with the whole liver/tumour interface profile, including immune infiltrate and micrometastases, it may gain a key role in clinical decision-making. As anticipated by the present data, radiomics could unveil the modifications of the liver–tumour interface under chemotherapy and the true effectiveness of systemic treatments, currently scarcely predicted by standard radiological parameters [38]. The longitudinal extension of peritumoural radiomic modification into the peritumoural tissue could help surgeons define the adequate margin width and, consequently, the adequate surgical procedure. Furthermore, quantitative indices (e.g., entropy load) could be devised by estimating the volume and the absolute voxel values of the high-entropy areas; these indices could be tested as predictors of tumour aggressiveness, therapy response, and survival. Parametric imaging modalities able to show the actual voxel-by-voxel entropy and uniformity variation in a PET-like overlay could make radiomics easy and accessible to clinicians. The entropy map of the tumour that we recently proposed could be extended to the peritumoural tissue [39].

This study has a few limitations. Firstly, it is a retrospective analysis of a limited population, thus, possibly subject to a selection bias. Furthermore, despite implementing a standardised protocol, differences in the evaluated CT data may still exist due to the bi-institutional design, which includes different devices and the 12-year enrolment period. A larger population in a prospective setting would allow a more detailed analysis of the subgroups and ensure greater robustness and broader data generalizability. Nevertheless, the present data are coherent with previous pathology analyses on peritumoural tissue [13,34]. Secondly, we do not have information beyond the first peritumoural centimetre. Further, VOIs were difficult to draw because many lesions are close to the liver capsule, major blood vessels, or other tumours. Even if a broader analysis would be of interest, the maximal recommended margin width is 1 cm, and the pathology studies reported microsatellites within the first 4–5 peritumoural millimetres [13,14]. Thirdly, the most appropriate study to determine the impact of chemotherapy on the peritumoural tissue would involve comparing paired CT scans before and after treatment. This was not feasible in the present series due to the limited availability of paired CT scans suitable for radiomic analysis. Such comparative studies should be the focus of future prospective analyses. Lastly, a correlation analysis between variation in radiomic and pathology or surgical data is lacking. This analysis is recommended but should rely on prospective data (for the pathology analyses) or consider all predictive factors of local recurrence. Such analyses are planned but are far beyond the scope of the present study.

## 5. Conclusions

The radiomic analysis of the liver parenchyma surrounding colorectal liver metastases unveiled a wide halo of progressively decreasing entropy and increasing uniformity despite a normal radiological aspect. Modifications are less pronounced in small metastases and patients responding to chemotherapy. The radiomic gradient of the peritumoural tissue needs to be investigated at a pathological level. Still, it could become a relevant biomarker driving clinical decision processes of both medical and surgical oncologists.

## Figures and Tables

**Table 1:** Patient characteristics.

Per-Patient Data	
Number of patients	51
Sex M:F	37 (73%):14 (27%)
Age, years	64 (40–76)
Number of metastases	1 (1–3)
Metastases size, mm	33 (11–110)
>50 mm	6 (12%)
Pre-treatment chemotherapy	29 (57%)
>1 line	2
Oxaliplatin	18
Irinotecan	10
Oxaliplatin + Irinotecan	1
Associated anti-VEGF targeted therapy	15
Associated anti-EGFR targeted therapy	8
Number of cycles	8 (4–18)
Partial response/Stable disease	22/7
**Per-Lesion Data**	
Number of tumours	63
Metastases size, mm	30 (10–110)
>50 mm	6 (10%)
Volume, voxel	6.07 × 10^3^ (0.33 × 10^3^–69.87 × 10^3^)
Pre-treatment chemotherapy	37 (59%)
Partial response/Stable disease *	29/8

Mean (range) or number (%). * According to the RECIST v1.1 and morphologic criteria [20,22].

**Table 2:** Absolute values of the analysed indices (HU-mean, entropy, and uniformity) and their delta (value and percentage) with the value in the virtual biopsy within different VOIs. The values are reported as mean ± standard deviation if the variable has a normal distribution and as median (IQR) if otherwise.

	Absolute Value	Delta Value with Virtual Biopsy	Delta % with Virtual Biopsy
Hu-mean			
Tumour	74.8 ± 17.3	−28.2 (−44.5–−20.5)	−27.7% (−38.8%–−21.5%)
1 mm	97.8 ± 15.4	−9.5 ± 10.2	−9.1% (−14.2%–−2.1%)
2 mm	106.1 ± 17.4	−2.4 (−7.3–+4.5)	−2.2% (−7.4%–+4.2%)
3–4 mm	110.1 ± 17.6	+1.7 (−2.2–+7.6)	+1.5% (−1.6%–+7.0%)
5–6 mm	110.5 ± 18.2	+3.2 ± 7.4	+2.0% (−1.8%–+7.3%)
7–8 mm	109.9 ± 18.6	+2.6 ± 6.4	+1.9% (−2.5%–+6.4%)
9–10 mm	109.2 ± 18.6	+2.0 ± 5.8	+2.0% ± 5.8%
Virtual biopsy	107.3 ± 18.1	-	-
Entropy (log2)			
Tumour	3.11 ± 0.33	+0.50 (+0.30–+0.85)	+19.1% (+12.1%–36.5%)
1 mm	3.02 ± 0.36	+0.49 (+0.27–+0.68)	+19.5% (+11.1%–+25.8%)
2 mm	2.90 ± 0.41	+0.38 ± 0.32	+14.3% (+5.0%–+23.7%)
3–4 mm	2.80 ± 0.40	+0.28 ± 0.28	+10.4% (+2.4%–+19.1%)
5–6 mm	2.76 ± 0.41	+0.24 ± 0.27	+10.2% ± 11.2%
7–8 mm	2.72 ± 0.40	+0.19 ± 0.24	+8.2% ± 10.0%
9–10 mm	2.71 ± 0.36	+0.18 ± 0.21	+8.0% ± 9.4%
Virtual biopsy	2.54 (2.32–2.80)	-	-
Uniformity			
Tumour	0.139 ± 0.033	−0.059 (−0.098–−0.037)	−32.4% ± 16.0%
1 mm	0.149 ± 0.036	−0.059 (−0.073–−0.035)	−28.0% ± 14.3%
2 mm	0.167 ± 0.044	−0.040 (−0.067–−0.015)	−20.1% ± 16.7%
3–4 mm	0.176 (0.144–0.202)	−0.033 ± 0.037	−14.0% ± 15.5%
5–6 mm	0.183 (0.151–0.201)	−0.027 ± 0.034	−11.7% ± 15.3%
7–8 mm	0.185 (0.151–0.216)	−0.023 ± 0.032	−10.1% ± 14.5%
9–10 mm	0.187 (0.157–0.204)	−0.024 ± 0.030	−9.9% ± 12.8%
Virtual biopsy	0.199 (0.176–0.234)	-	-

**Table 3:** Comparative analysis of the absolute values of the analysed indices (HU-mean, entropy, and uniformity) within different VOIs: comparison with the contiguous VOI (first column), Tumour-VOI (second column), and virtual biopsy (third column).

	*p*-Value vs. Previous VOI	*p*-Value vs. Tumour	*p*-Value vs. Virtual Biopsy
Hu-mean			
Tumour	-	-	**<0.001**
1 mm	**<0.001**	**<0.001**	**0.002**
2 mm	**0.006**	**<0.001**	0.709
3–4 mm	0.203	**<0.001**	0.378
5–6 mm	0.894	**<0.001**	0.320
7–8 mm	0.847	**<0.001**	0.427
9–10 mm	0.846	**<0.001**	0.550
Virtual biopsy	0.550	**<0.001**	-
Entropy (log2)			
Tumour	-	-	<0.001 *
1 mm	0.153	0.153	<0.001 *
2 mm	0.078	**0.002**	<0.001 *
3–4 mm	0.176	**<0.001**	<0.001 *
5–6 mm	0.603	**<0.001**	<0.001 *
7–8 mm	0.520	**<0.001**	0.006 *
9–10 mm	0.874	**<0.001**	0.003 *
Virtual biopsy	0.003 *	<0.001 *	-
Uniformity			
Tumour	-	-	<0.001 *
1 mm	0.091	0.091	<0.001 *
2 mm	0.017	**<0.001**	<0.001 *
3–4 mm	0.159 *	<0.001 *	<0.001 *
5–6 mm	0.553 *	<0.001 *	0.002 *
7–8 mm	0.705 *	<0.001 *	0.010 *
9–10 mm	0.938 *	<0.001 *	0.007 *
Virtual biopsy	0.007 *	<0.001 *	-

* Mann–Whitney U test. According to the Bonferroni correction for multiple comparisons, a *p*-value ≤ 0.007 was considered significant (bold text).

## Data Availability

The dataset used to elaborate the presented data can be obtained by the corresponding author on a reasonable request.

## Supplementary Materials

The following supporting information can be downloaded at https://www.mdpi.com/article/10.3390/diagnostics14141552/s1, Supplementary Figure S1. Delta percentage values of the HU-mean within various VOIs (tumor and rims) against those of the non-tumoral parenchyma (virtual biopsy); Supplementary Figure S2. Delta percentage values of the entropy within different VOIs (tumor and rims) against those of the non-tumoral parenchyma (virtual biopsy); Supplementary Figure S3. Delta percentage values of the uniformity within different VOIs (tumor and rims) against those of the non-tumoral parenchyma (virtual biopsy); Supplementary Figure S4. Entropy and uniformity values according to the tumor size (10–30 mm vs. >30 mm) VB: virtual biopsy; Supplementary Figure S5. Entropy and uniformity values according to the administration of preoperative chemotherapy (no chemotherapy vs. chemotherapy with partial response). PR: partial response; VB: virtual biopsy.
