# Supplementary material for: Radiomic Gradient in Peritumoural Tissue of Liver Metastases: A Biomarker for Clinical Practice? Analysing Density, Entropy, and Uniformity Variations with Distance from the Tumour
Source: Diagnostics (Basel). 2024 Jul 18;14(14):1552. doi: 10.3390/diagnostics14141552 (PMC11276558; doi:10.3390/diagnostics14141552)

Supplementary Figure S1. Delta percentage values of the HU-mean within various VOIs (tumor and rims) against those of the non-tumoral parenchyma (virtual biopsy).

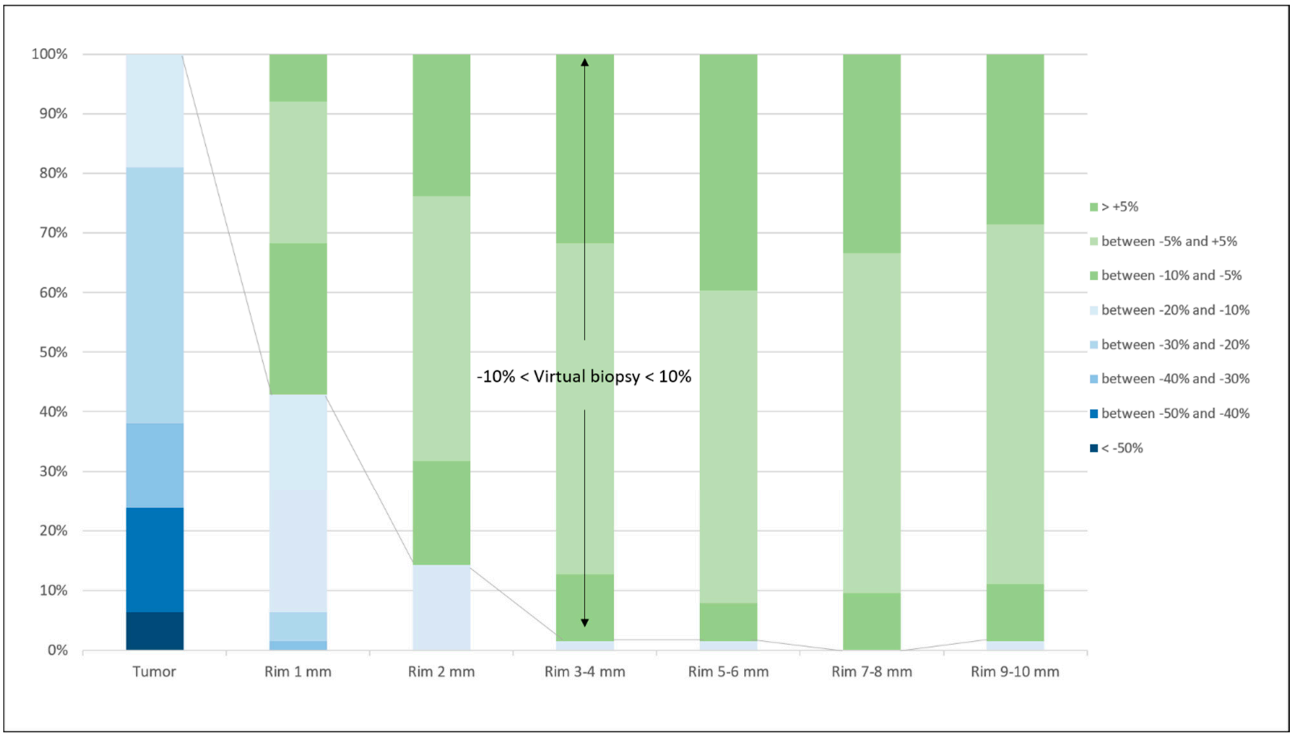

Supplementary Figure S2. Delta percentage values of the entropy within different VOIs (tumor and rims) against those of the non-tumoral parenchyma (virtual biopsy).

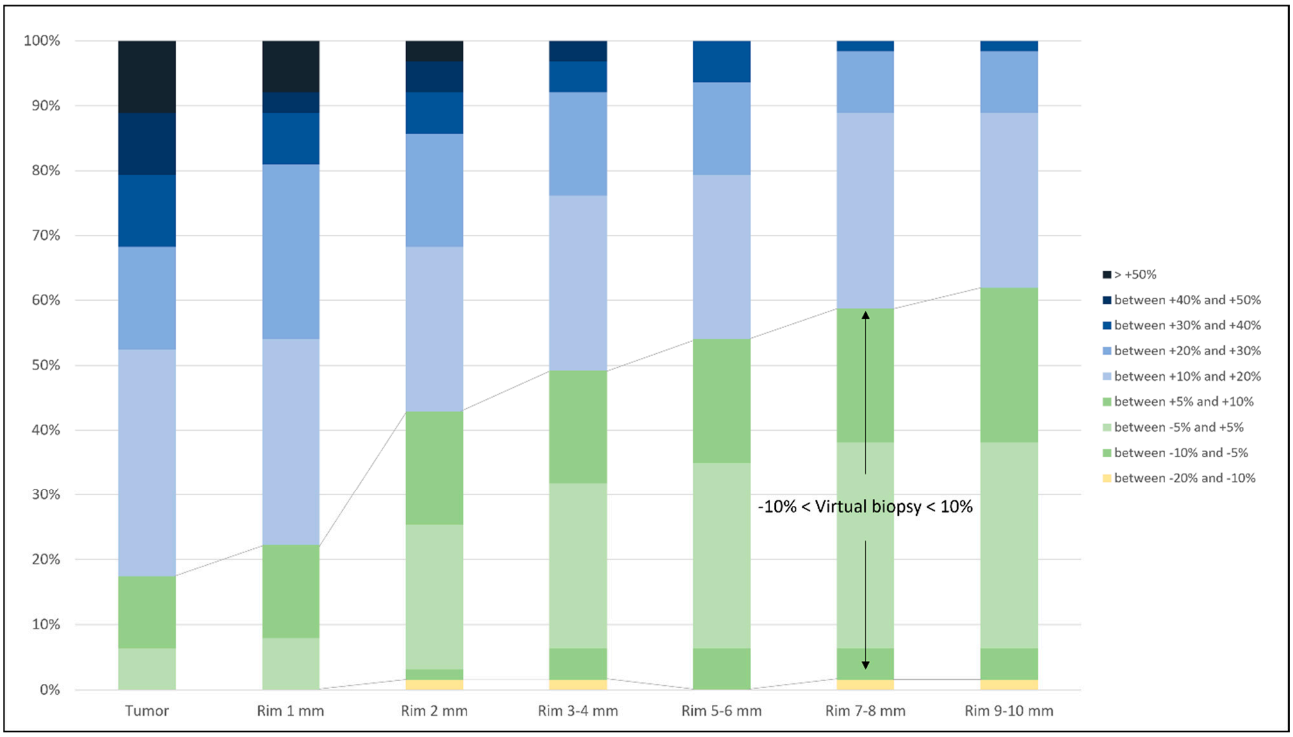

Supplementary Figure S3. Delta percentage values of the uniformity within different VOIs (tumor and rims) against those of the non-tumoral parenchyma (virtual biopsy).

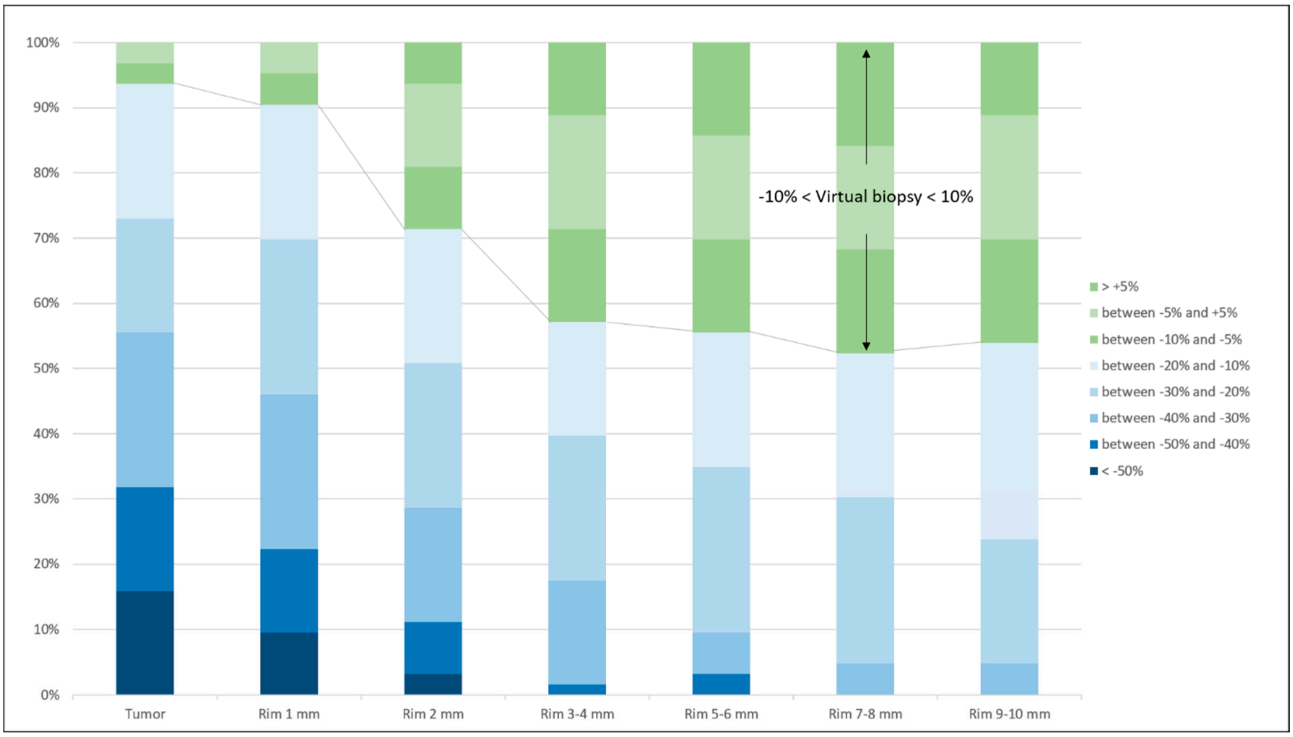

Supplementary Figure S4. Entropy and uniformity values according to the tumor size (10-30 mm vs. >30 mm) *VB: virtual biopsy*

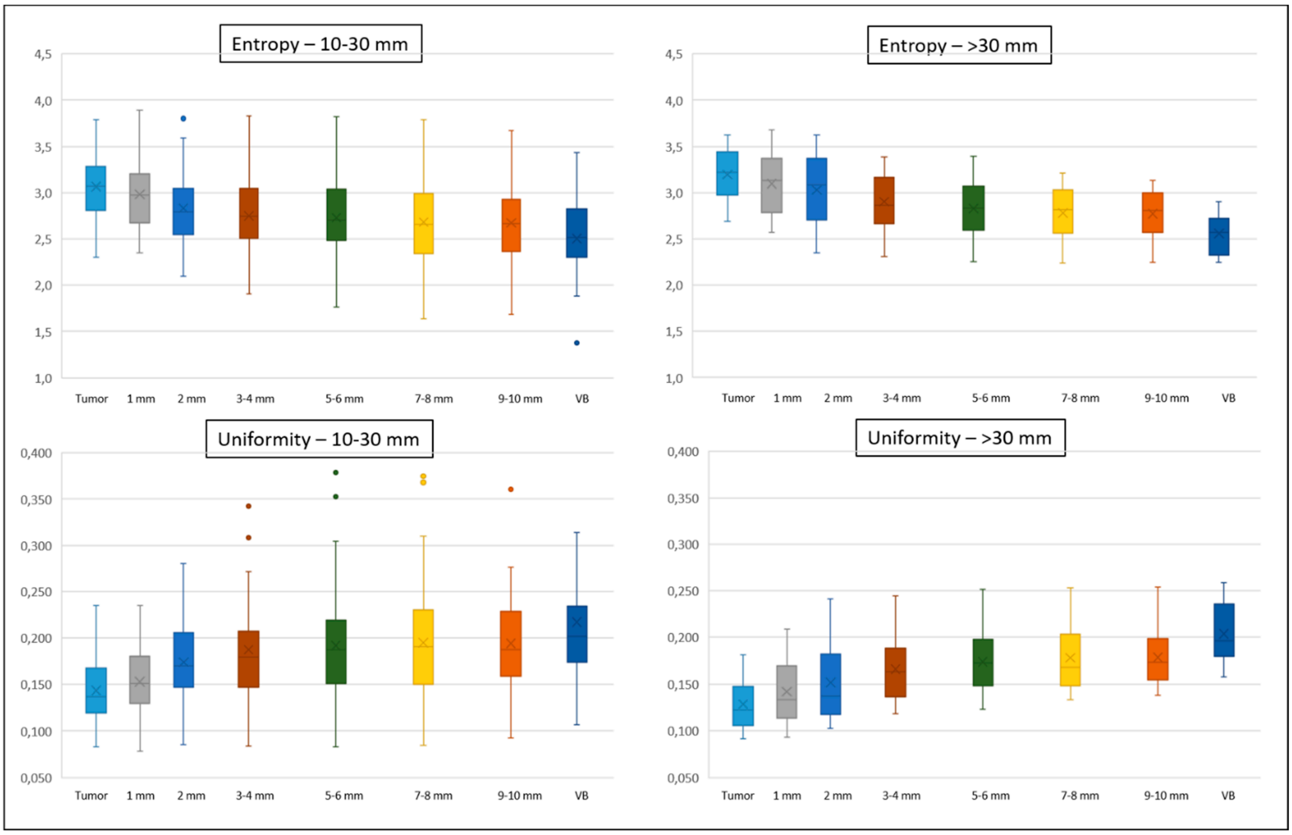

Supplementary Figure S5. Entropy and uniformity values according to the administration of preoperative chemotherapy (no chemotherapy vs. chemotherapy with partial response). *PR: partial response; VB: virtual biopsy*

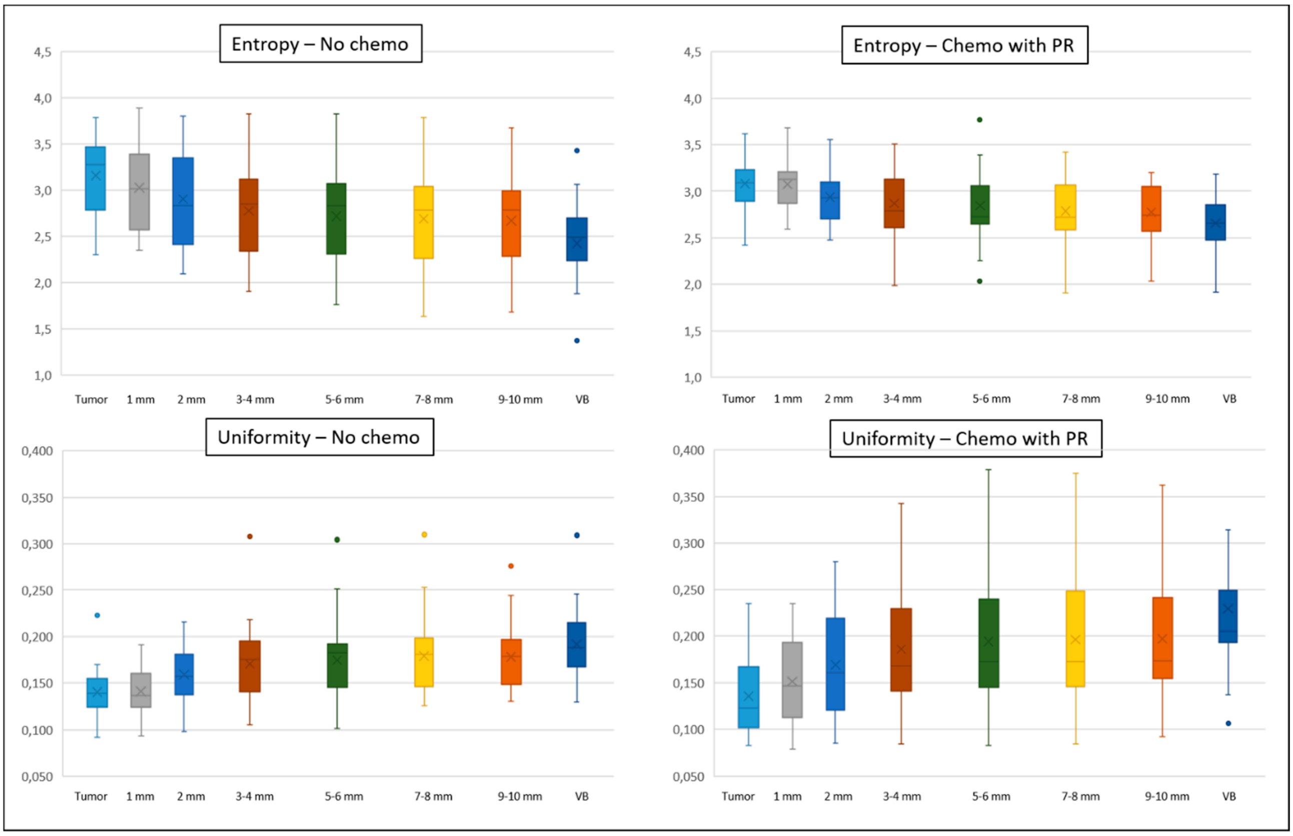

Supplement: Supplementary file 1 [file diagnostics-14-01552-s001.zip › diagnostics-3022164-supplementary.pdf]
